# Ciprofloxacin-resistant *Escherichia coli* in Central Greece: mechanisms of resistance and molecular identification

**DOI:** 10.1186/1471-2334-12-371

**Published:** 2012-12-23

**Authors:** Angeliki Mavroidi, Vivi Miriagou, Apostolos Liakopoulos, Εva Tzelepi, Angelos Stefos, George N Dalekos, Efthymia Petinaki

**Affiliations:** 1 Department of Microbiology, University Hospital of Larissa, Larissa, Greece; 2 Laboratory of Bacteriology, Hellenic Pasteur Institute, Athens, Greece; 3 Department of Medicine, Medical School, University of Thessaly, Larissa, Greece; 4 Department of Microbiology, Medical School, University of Thessaly, Biopolis, Larissa, Greece

**Keywords:** *Escherichia coli*, Quinolones,MLST, Beta lactamases

## Abstract

**Background:**

Fluoroquinolone resistant *E. coli* isolates, that are also resistant to other classes of antibiotics, is a significant challenge to antibiotic treatment and infection control policies. In Central Greece a significant increase of ciprofloxacin-resistant *Escherichia coli* has occurred during 2011, indicating the need for further analysis.

**Methods:**

A total of 106 ciprofloxacin-resistant out of 505 *E. coli* isolates consecutively collected during an eight months period in a tertiary Greek hospital of Central Greece were studied. Antimicrobial susceptibility patterns and mechanisms of resistance to quinolones were assessed, whereas selected isolates were further characterized by multilocus sequence typing and β-lactamase content.

**Results:**

Sequence analysis of the quinolone-resistance determining region of the *gyrA* and *parC* genes has revealed that 63% of the ciprofloxacin-resistant *E. coli* harbored a distinct amino acid substitution pattern (GyrA:S83L + D87N; ParC:S80I + E84V), while 34% and 3% carried the patterns GyrA:S83L + D87N; ParC:S80I and GyrA:S83L + D87N; ParC:S80I + E84G respectively. The *aac (6’)-1b-cr* plasmid-mediated quinolone resistance determinant was also detected*;* none of the isolates was found to carry the *qnrA*, *qnrB* and *qnrS*.

Genotyping of a subset of 35 selected ciprofloxacin-resistant *E. coli* by multilocus sequence typing has revealed the presence of nine sequence types; ST131 and ST410 were the most prevalent and were exclusively correlated with hospital and health care associated infections, while strains belonging to STs 393, 361 and 162 were associated with community acquired infections. The GyrA:S83L + D87N; ParC:S80I + E84V substitution pattern was found exclusively among ST131 ciprofloxacin-resistant *E. coli*. Extended-spectrum β-lactamase-positive ST131 ciprofloxacin-resistant isolates produced CTX-M-type enzymes; eight the CTX-M-15 and one the CTX-M-3 variant. CTX-M-1 like and KPC-2 enzymes were detected in five and four ST410 ciprofloxacin-resistant *E. coli* isolates, respectively.

**Conclusions:**

Our findings suggest that, ST131 and ST410 predominate in the ciprofloxacin resistant *E. coli* population.

## Background

*Escherichia coli* is among the major pathogens in both community and hospital settings [1]. The prevalence of multidrug-resistant *E. coli*, (i.e., *E. coli* isolates resistant to more than three classes of antimicrobial agents) has been increased worldwide in the past decades. The emergence and worldwide dissemination of fluoroquinolone resistant *E. coli* isolates, that are also resistant to newer β-lactams due to the production of extended-spectrum β-lactamases (ESBLs) particularly CTX-M-type enzymes, is a significant challenge to antibiotic treatment and infection control policies [1]. The application of multilocus sequence typing (MLST) to isolates producing CTX-M-15 ESBL led to the recognition of an internationally disseminated clone, ST131, which is a virulent phylogroup B2, uropathogenic *E. coli* lineage. ST131 *E. coli* is associated with resistance to fluoroquinolones and aminoglycosides.

Quinolones are widely used antimicrobials for the treatment of bacterial infections [2]. Their wide use has triggered increased bacterial resistance worldwide. Mutations in *gyrA* and *parC* genes are the most common mechanism involved in high-level quinolone resistance, yet the spread of plasmid- mediated quinolone resistance genes and efflux-pump mutants have also been described. In Greece, according to the recent data of WHONET the rate of ciprofloxacin resistant *(*CIP-R) *E. coli* varied from hospital to hospital and ranged from 5.6 to 49.5%. In the University Hospital of Larissa (UHL), that is the main tertiary hospital of Central Greece and serves a region of 1,000,000 habitants, an increase of ciprofloxacin-resistant *E. coli* from 16.3% in 2010 to 21% in 2011 was recorded. The aim of this study was to assess the epidemiological traits, mechanisms of resistance to fluoroquinolones, phylogenetic relationship and co-existing mechanism of resistance to newer β-lactams of CIP-R *E. coli* strains isolated in our institution during 2011.

## Methods

### Bacterial isolates,susceptibility testing and clinical data

From May to December 2011, a total of 505 consecutive *E. coli* isolates were recovered from clinical samples taken as part of standard care of an equal number of individual patients admitted to the University Hospital of Larissa, Central Greece. Identification and antimicrobial susceptibility testing of bacterial isolates was firstly performed by the Vitek-2 system (bioMérieux, Marcy l’Étoile, France). The interpretive criteria of the European Committee on Antimicrobial Susceptibility Testing were used (http://eucast.org/clinical_breakpoints).

Determination of MICs to ciprofloxacin norfloxacin, imipenem and meropenem was performed by the Etest method (bioMérieux). The double disk synergy test (DDST) was used to determine the ESBL production, as described previously [3]. The modified Hodge test was used for phenotypic detection of carbapenemase production [3]. All *E. coli,* that were classified as resistant to ciprofloxacin (MICs > =4 mg/L), were further analyzed for the underlying mechanisms of quinolone resistance, molecular typing and β-lactamase content.

The medical records of patients diagnosed with CIP-R *E. coli* infection were reviewed regarding their current and previous hospitalizations. Before obtaining the clinical information of the patients, approval was received by the Ethics Committee of the UHL, that is represented by the Infection Control Committee (number of permission 1234). Infections developed 48 h after hospital admission were characterized as UHL hospital-acquired infections. Infections due to CIP-R *E. coli* diagnosed within 48 h of hospital admission were characterized as community acquired infections. Finally, infections in patients who had been hospitalized in the preceding 6 months for more than 48 h in hospital facilities or nursing homes such as infections in patients transmitted to UHL from other hospitals were considered as health care associated infections.

### PCR and sequencing of the Quinolone Resistance-Determining Region (QRDR) of the *gyrA* and *parC* genes

All Cip-R *E. coli* were screened for the presence of mutations in the QRDR of the *gyrA* and *parC* genes. After DNA extraction by using the Quick-gDNA ^TM^ MiniPrep kit (ZYMO RESEARCH Corp., USA), the QRDRs of both *gyrA* and *parC* genes were amplified by polymerase chain reaction (PCR), as described previously [4] and the amplicons were sequenced on both DNA strands using an ABI3730 DNA sequencer (Applied Biosystems, Warrington, United Kingdom). For each isolate, the sequences of the *gyrA* and *parC* gene fragments were concatenated, maintaining the +1 reading frame, aligned and a neighbor-joining tree was constructed from the aligned sequences using the MEGA software [5].

### Detection of plasmid-mediated quinolone resistance genes *qnrA*, *qnrB, qnrS* and *aac(6’)-Ib-cr* variant

Primers and conditions for PCR amplification of the *qnrA*, *qnrB* and *qnrS* genes, which encode three target- protecting proteins and the *aac(6’)-Ib-cr* variant, which encodes a bifunctional aninoglycoside- fluoroquinolone modified enzyme, were used as described previously [6], and the amplified PCR products obtained were sequenced on both DNA strands as described previously.

### Detection of beta-lactamases

Detection of the *bla* genes was performed by PCR using a panel of specific primers for *bla*_TEM-1_, *bla*_OXA-1_, *bla*_SHV_, *bla*_CTX-M_, *bla*_CMY_, *bla*_VIM_ and *bla*_KPC_, as described previously [7]. PCR products were purified by using the PureLink™ PCR Purification Kit (Invitrogen, USA) kit and sequenced. Nucleotide and deduced protein sequences were identified by comparing the sequences of the database of G. Jacoby and K. Bush (http://www.lahey.org/Studies).

### Molecular typing of isolates

Τhe major phylogenetic groups (A, B1, B2, D) were determined by PCR amplification of the three gene fragments of the scheme (*chuA*, *yjaA* and TSPE4.C2). Phylogroups were determined as described previously [8]. MLST was performed by PCR amplification and sequencing of seven housekeeping genes: *adk* (adenylate kinase), *fumC* (fumarate hydratase), *gyrB* (DNA gyrase), *icd* (isocitrate/ isopropylmalate dehydrogenase), *mdh* (malate dehydrogenase), *purA* (adenylosuccinate dehydrogenase), *recA* (ATP/GTP binding motif) by using primers and conditions as described at the MLST Databases at the ERI, University College Cork [9]; http://mlst.ucc.ie/mlstdbs/E.coli. Sequences were obtained on both DNA strands, and alleles and STs were compared with those assigned at the MLST website. Non overlapping groups of related STs were identified using eBURST, with the default setting for the definition of groups [10]; http://eburst.mlst.net.

## Results

One hundred six out 505 (21%) *E. coli* isolates were found to be resistant to quinolones. These isolates were obtained from various clinical specimens; mainly from urine (76 out of 106; 72%) and blood (12 out of 106; 11%), but also from bronchial secretions, cutaneous lessions and sputum. Among them, 30% (32 isolates) were recovered from community acquired infections, 15% (16 isolates) from UHL hospital acquired infections and 55% (58 isolates) from health care associated infections.

Among 106 CIP-R *E. coli* isolates, 57% showed an ESBL phenotype, exhibiting resistance to penicillins, expanded- spectrum cephalosporins and aztreonam, while, 4.7% exhibited also resistance to at least one carbapenem, 72% were resistant to trimethoprim- sulfamethoxazole, 70% to tetracycline, 60% to tobramycin, whereas, only 25% to gentamicin. Nineteen percent of the CIP-R *E. coli* isolates exhibited concurrent resistance to four other classes of antimicrobial agents, 58.5% to three classes, 13.2% to two classes and 3.8% to one class; the remaining 5.5% showed resistance only to quinolones.

Sequencing of the QRDR regions of the *gyrA* and *parC* genes of CIP-R isolates has revealed three different amino acid substitution patterns: GyrA:S83L + D87N; ParC:S80I + E84V (n = 67, 63%), GyrA:S83L + D87N; ParC:S80I (n = 36, 34%) and GyrA:S83L + D87N; ParC:S80I + E84G (n = 3, 3%). All but one of the 67 CIP-R *E. coli* isolates, that possessed the GyrA:S83L + D87N; ParC:S80I + E84V pattern, showed identical nucleotide sequences in the *gyrA* and *parC*, with the exception showing only a synonymous nucleotide substitution in the *parC* gene. In addition, the three isolates with the GyrA:S83L + D87N; ParC:S80I + E84G pattern had identical nucleotide sequences. On the contrary, the nucleotide sequences of *gyrA*/ *parC* gene fragments of the 36 CIP-R isolates with the GyrA:S83L + D87N;ParC:S80I substitution pattern were found to be more polymorphic.

Out of the 106 CIP-R *coli* isolates, 35 were selected for further investigation including 21 with the pattern GyrA:S83L + D87N; ParC:S80I + E84V, 13 with the pattern GyrA:S83L + D87N; ParC:S80I, and one with the pattern GyrA:S83L + D87N; ParC:S80I + E84G. The selection was designed so as to include proportionally all the variations in the amino and nucleotide acid substitution patterns observed in the GyrA and ParC, the antimicrobial resistance patterns, the origin and the distribution of the isolates over the study period (Table 1).

**Table 1:** **Microbiological characteristics of the 35 representative CIP-R *E. coli; *phylogroups, MLST STs (clonal complexes; Cplx), presence/absence of the *aac (6’)-1b-cr *variant and beta-lactamase content**

**Strain ID**	**Isolation date**	**Antimicrobial resistant pattern**	**ESBL**	**Phylogroup/ MLST ST (CC)**	***aac (6’)-Ib-cr***	**beta-lactamase content**
**GyrA:S83L** + **D87N/ ParC: S80I** + **E84V (n = 21)**						
347	13/6/2011	AM,AMC,CAZ,CTX,ATM,GM,NN,TE,SXT,CIP, NOR	positive	B2/ ST131	( − )	CTX-M-15
161	27/5/2011	AM,AMC,CAZ,CTX,ATM, NN,TE,SXT,CIP NOR	positive	B2/ ST131	( + )	CTX-M-15 + OXA-1
391	30/5/2011	AM,AMC,CAZ,CTX,ATM,GM,NN,TE,SXT,CIP,NOR	positive	B2/ ST131	( + )	CTX-M-15 + OXA-1
392	9/6/2011	AM,AMC,CAZ,CTX,ATM,GM,NN,TE,SXT,CIP,NOR	positive	B2/ ST131	( + )	CTX-M-15 + OXA-1
393	18/7/2011	AM,AMC,CAZ,CTX,ATM,GM,NN,TE,SXT,CIP,NOR	positive	B2/ ST131	( + )	CTX-M-15 + OXA-1
399	2/8/2011	AM,AMC,CAZ,CTX,ATM,GM,NN,TE,SXT,CIP,NOR	positive	B2/ ST131	( + )	CTX-M-15 + OXA-1
160	3/5/2011	AM,AMC,CAZ,CTX,ATM,SXT,CIP,NOR	positive	B2/ ST131	( + )	CTXM-15 + TEM-1
505	4/9/2011	AM,AMC,CAZ,CTX,ATM,AN,GM,NN,SXT,CIP,NOR	positive	B2/ ST131	( + )	CTX-M-15 + TEM-1 + OXA-1
397	4/8/2011	AM,AMC,CAZ,CTX,ATM,TE, SXT, CIP, NOR	positive	B2/ ST131	( − )	CTX-M-3
270	1/5/2011	AM,AMC,NN,SXT,CIP,NOR	negative	B2/ ST131	( + )	OXA-1
301	5/5/2011	AM, AMC,NN,TE,CIP,NOR	negative	B2/ ST131	( + )	OXA-1
307	6/6/2011	AM,AMC, GM, NN, TE,CIP, NOR	negative	B2/ ST131	( + )	OXA-1
252	1/5/2011	AM, SXT,CIP,NOR	negative	B2/ ST131	( − )	TEM-1
206	16/5/2011	AM,AMC,GM,TE,SXT,CIP,NOR	negative	B2/ ST131	( + )	TEM-1 + OXA-1
320	25/5/2011	AM,AMC,CIP,NOR	negative	B2/ ST131	( + )	TEM-1 + OXA-1
346	3/6/2011	AM,AMC,AN,GM,NN,SXT,TE,CIP,NOR	negative	B2/ ST131	( + )	TEM-1 + OXA-1
803	4/12/2011	AM,AMC,CAZ,CTX, IMP,MEM,CIP, NOR	negative	B2/ ST131	( + )	VIM-1-like + TEM-1+ OXA-1
220	5/5/2011	TE, SXT,CIP,NOR	negative	B2/ ST131	( − )	none
321	18/7/2011	CIP,NOR	negative	B2/ ST131	( − )	none
354	1/7/2011	TE,CIP,NOR	negative	B2/ ST131	( − )	none
355	15/8/2011	TE,SXT,CIP,NOR	negative	B2/ ST131	( − )	none
**GyrA:S83L** + **D87N/ ParC: S80I** + **E84G (n = 1)**						
296	6/8/2011	AM, TE, CIP, NOR	negative	D/ ST393 (ST31 Cplx)	( + )	TEM-1
**Strain ID**	**Isolation date**	**Antimicrobial susceptibility pattern**	**ESBL**	**Phylogroup/MLST ST (CC)**	***aac (6’)-Ib-cr***	**beta-lactamase content**
**GyrA:S83L** + **D87N/ ParC: S80I (n = 13)**						
148	9/6/2011	AM,AMC,CAZ,CTX,ATM,IMP,MEM,NN,SXT,CIP,NOR	positive	B2/ ST410	( + )	KPC-2 + CTXM-15 + TEM-1 + OXA-1
				(ST23 Cplx)		
182	17/5/2011	AM,AMC,CAZ,CTX,ATM,IMP,MEM,AN,NN,TE,CIP,NOR	positive	B2/ ST410	( + )	KPC-2 + CTXM-3 + TEM-1+ OXA-1
				(ST23 Cplx)		
252	10/6/2011	AM,AMC,CAZ,CTX,ATM,IMP,MEM,NN,TE,CIP,NOR	positive	B2/ ST410	( + )	KPC-2 + CTXM-3 + TEM-1 + OXA-1
				(ST23 Cplx)		
648	31/12/2011	AM,AMC,CAZ,CTX,IMP,MEM,NN,TE,CIP,NOR	negative	B2/ ST410	( − )	KPC-2 + TEM-1
				(ST23 Cplx)		
132	24/5/2011	AM,AMC,CAZ,CTX,ATM,AN,NN,TE,SXT,CIP,NOR	positive	B2/ ST410	( + )	CTXM-15 + TEM-1 + OXA-1
				(ST23 Cplx)		
281	15/8/2011	AM,AMC,CAZ,CTX,ATM,AN,GM,NN,TE,SXT,CIP,NOR	positive	B2/ ST410	( + )	CTXM-15 + OXA-1
				(ST23 Cplx)		
384	30/11/2011	AM,AMC,CAZ,CTX,ATM,AN,TE,SXT,CIP,NOR	positive	B2/ ST44	( + )	CTXM-15
				(ST10 Cplx)		
383	22/9/2011	AM,TE,SXT,CIP,NOR	negative	A/ ST90	( + )	TEM-1
				(ST23 Cplx)		
259	14/7/2011	AM,TE,SXT,CIP,NOR	negative	B1/ ST162	( + )	TEM-1
				(ST469 Cplx)		
328	10/9/2011	AM,SXT,CIP,NOR	negative	B2/ ST361	( − )	TEM-1
362	21/10/2011	AM,TE,SXT,CIP,NOR	negative	D/ ST393	( − )	TEM-1
				(ST31 Cplx)		
129	25/5/2011	AM,TE,SXT,CIP,NOR	negative	B2/ ST1140	( − )	TEM-1
327	9/6/2011	CIP, NOR	negative	A/ ST2509	( − )	none

The 35 representative CIP-R *E.coli* isolates were distributed into phylogroups B2 (30 isolates), D (two isolates), A (two isolates) and B1 (one isolate) [Table 1]. Genotyping by MLST has revealed the presence of nine STs. ST131 (21 out 35 isolates), and ST410 (six out 35 isolates) were the most prevalent; ST44, ST90, ST162, ST361, ST1140, ST2509 included one isolate, while, ST393 included two isolates. According to clinical data, all ST131 isolates were associated with UHL hospital acquired infections and health care associated infections, ST410 CIP-R *E. coli* were exclusively associated with health care associated infections, while STs 393, 361 and 162 from community acquired infections. Strains that belonged to ST90, 44, 1140 and 2509 were isolated from patients with UHL hospital acquired infections.

**Figure 1:**
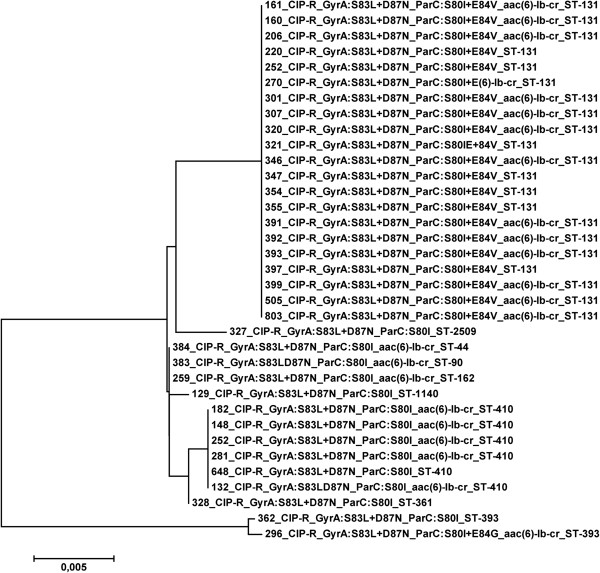
**Neighbor- joining tree of the 35 representative CIP-R *E. coli; *for each isolate, the GyrA/ParC amino acid substitution pattern, the presence/absence of the *aac (6’)-1b-cr *variant and the MLST ST is indicated.**

Although, only three amino acid substitution patterns in the GyrA/ParC were identified among the CIP-R *E. coli*, we have sought to investigate any possible correlations of the nucleotide polymorphisms and the STs of the isolates. For this purpose, a neighbor-joining tree was constructed from the concatenated sequences of the *gyrA*/ *parC* gene fragments (Figure 1). The pattern GyrA:S83L + D87N;ParC:S80I + E84V was associated with ST131 strains possessing identical nucleotide sequences, while, the GyrA:S83L + D87N; ParC:S80I was found to various STs, including ST410. Except from one isolate (ID 362), the rest isolates with this substitution pattern differed in 12 polymorphic sites of the 643 bp nucleotide sequence of the *gyrA*/*parC* fragments. The third pattern, GyrA:S83L + D87N; ParC:S80I + E84G, was associated with ST393 (ID 296). The isolate ID 362, that belonged also to ST393, showed similarity in the *gyrA*/ *parC* nucleotide sequences with the isolate ID 296 and they differed at only a single nucleotide site.

The presence of plasmid- mediated quinolone resistance genes was also investigated in the 35 CIP-R *E.coli* isolates (Table 1, Figure 1). The *aac (6’)-1b-cr* variant was detected in 23 out of 35 CIP-R *E.coli*; of these 14 were ST131 and five ST410. The remaining four isolates belonged to ST44, ST90 and ST162 and ST393. None isolate was found to carry any of the *qnrA, qnrB* and *qnrS* gene.

The β-lactamase content of the 35 Cip-R *E. coli* was also determined (Table 1). ESBL- positive CIP-R isolates were found to produce enzymes of the CTX-M-1 family (n = 15); CTX-M-15 (n = 12) and CTX-M-3 (n = 3). Among them, nine belonged to ST131, five to ST410 and one to ST44 (Table 1). One out of the twelve ESBL-negative ST131 was a VIM-1 producer. Three ST410 CTX-M-producing strains co-produced the KPC-2 carbapenemase.

## Discussion

During an eight months period of 2011, CIP-*R E. coli* accounted for 21% of the total *E. coli* isolates recovered from various clinical specimens from outpatients and inpatients of the UHL. The majority of the CIP-R *E. coli* isolates were multidrug-resistant, posing a challenge for therapeutic options. Only 5.5% were resistant to fluoroquinolones, but susceptible to various antimicrobial classes. The latter strains were recovered from both community and hospital acquired infections.

The majority of CIP-R *E. coli* belonged to ST131 and ST410, which were recovered from hospital and health care associated infections, whereas other studies have shown that such strains were disseminated in the community [11-16]. We have also shown previously that ST410 was linked with an outbreak of KPC- producing *E. coli* in a long-term care facility unit of Thessalia [17]; this clone, apart from carbapenemase producers, includes also isolates with CTX-M-15, as it was firstly reported in Spain [16]. All ST131 and ST410 CIP-R *E. coli* belonged to the virulent phylogroup B2, which is associated epidemiologically and experimentally with extraintestinal virulence [12,18]. The remaining isolates, that were isolated in a sporadic fashion in our study, were distributed equally to various STs, which have been previously reported among Cip-R *E. coli* isolates, and originated from community and hospital acquired infections [15].

In Greece, there are few studies on the mechanisms of resistance to fluoroquinolones [19-21], but to our knowledge this is the first report on the association of *gyrA* and *parC* mutations and the phylogenetic lineages of ciprofloxacin-resistant *E. coli* in Greece. According to our results, ST131 and ST410 carried specific patterns of GyrA/ParC amino substitutions. In more details, ST131 Cip-R *E. coli* in our hospital possessed the same amino acid substitution pattern GyrA:S83L + D87N; ParC:S80I + E84V, which has been previously identified among ST131 CIP-R *E. coli* isolated from humans and companion animals in the United States, United Kingdom, Australia and Korea [13-15]. On the other hand, the six ST 410 *E. coli* of our study carried the GyrA:S83L + D87N; ParC:S80I pattern, which has been identified in previous studies [22,23]. Nevertheless, the sequence types of the latter strains have not been determined.

The presence of plasmid-mediated resistance genes (*qnrA*, *qnrB* and *qnrS*) has also been reported in previous studies in Greece [19-21], but these genes were not detected in our collection of CIP-R *E. coli* isolates. On the other hand, the *aac (6’)-1b-cr* variant was detected mainly in strains belonging to both ST131 and ST410, a finding that was also previously described [13,16].

Since isolates possessing the GyrA:S83L + D87N; ParC:S80I + E84V comprised a high percentage (67%) among CIP-R *E. coli*, and the representative isolates of this group have been assigned to ST131, we assume that the increase of fluoroquinolone-resistance during 2011 can be attributed to the dissemination of ST131 strains. The source and the time of importation of these isolates are unknown. As mentioned previously, since ST131 was first described in 2008 [24], it has disseminated worldwide [11-15,23-26]. Several factors such as host-to-host or foodborne transmission or environmental contamination have been suggested to contribute in their dissemination; reservoirs of ST131 have been identified in food and water sources, in nursing home residents, companion animals and food sources [27-29].

Central Greece is a rural area, where fluoroquinolones are widely used in veterinary and poultry, but no data exist about the incidence of resistant *E. coli* of animal source. It would be interesting to investigate the mechanisms of resistance and genetic relatedness of such strains in future studies. These studies would elucidate the relationships between STs of isolates recovered from human and animals and the roots of transmission.

## Conclusions

In the present study we have characterized the mechanisms of resistance and explored the genetic relatedness of CIP-R *E. coli* recovered from community, hospital and health care associated infections in a tertiary care hospital in Central Greece. Our findings suggest that ST131 and ST410 predominate in the CIP-R *E. coli* population in our institution. The increase of resistance to fluoroquinolones observed during 2011 is attributed mainly to the wide dissemination of ST131 CIP-R. *E. coli.*

## Abbreviations

CIP-R: ciprofloxacin resistant; MLST: Multilocus sequence typing; ST: Sequence type; CC: Clonal complex; ESBL: Extended-spectrum β-lactamases; KPC: *Klebsiella pneumoniae* carbapenemase; QRDR: Quinolone Resistance-Determining Region; UHL: University Hospital of Larissa; DDST: Double disk synergy test; AM: Amoxicillin; AMC: Amoxicillin + clavulanic acid; CAZ: Ceftazidime; CTX: Cefotaxime; ATM: Aztreonam; GM: Gentamicin; NN: Tobramycin; AN: Amikacin; TE: Tetracycline; SXT: Trimethoprim-sulfomethoxazole; CIP: Ciprofloxacin; NOR: Norfloxacin; IMP: Imipenem; MEM: Meropenem.

## Competing interests

The authors declare that they have no competing interests.

## Authors’ contributions

EP, AM, and VM conceived and designed the study. AM wrote the first draft of the paper and other co-authors contributed to the final draft. EP and GND were responsible for conducting the study and managing the data. AS conducted the interpretation of data. Others participated in data analysis and data interpretation. All authors read and approved the final manuscript.

## Pre-publication history

The pre-publication history for this paper can be accessed here:

http://www.biomedcentral.com/1471-2334/12/371/prepub
